# Analysis of Nucleotide Alterations in the E6 Genomic Region of Human Papillomavirus Types 6 and 11 in Condyloma Acuminatum Samples from Brazil

**DOI:** 10.1155/2019/5697573

**Published:** 2019-05-02

**Authors:** Marina Carrara Dias, Bruna Stuqui, Paola Jocelan Scarin Provazzi, Cíntia Bittar, Natália Maria Candido, Renata Prandini Adum de Matos, Rodolfo Miglioli Badial, Caroline Measso do Bonfim, Patricia Pereira dos Santos Melli, Silvana Maria Quintana, José Antônio Cordeiro, Paula Rahal, Marilia de Freitas Calmon

**Affiliations:** ^1^Institute of Biosciences, Letters and Exact Sciences of São Paulo State University, São José do Rio Preto, SP, Brazil; ^2^Clinical Hospital of Faculty of Medicine of Ribeirão Preto, São Paulo University, Ribeirão Preto, SP, Brazil; ^3^Faculty of Medicine of Ribeirão Preto, São Paulo University, Ribeirão Preto, SP, Brazil; ^4^Faculty of Medicine of Rio Preto, São José do Rio Preto, SP, Brazil

## Abstract

Condyloma acuminata (CA), or genital warts, are benign proliferative epidermal or mucous lesions that are caused by infection with human papillomavirus (HPV), mainly the low-risk types 6 and 11. HPV variants are defined as viral sequences that share identity in the nucleotide sequence of the L1 gene greater than 98%. Based on this criterion, HPV6 and 11 variant lineages have been studied, and there are ongoing attempts to correlate these genetic variants with different clinical findings of infection. Therefore, the aims of this study were to detect variants and nucleotide alterations present in the E6 regions of HPV types 6 and 11 found in CA samples, to correlate the HPV presence with the clinical-pathological data of the patients, and to determine phylogenetic relationships with variants from other places in the world. The E6 regions of 25 HPV6 samples and 7 HPV11 samples from CA were amplified using PCR with specific primers. The products were ligated to a cloning vector and five colonies of each sample were sequenced to observe the nucleotide alterations. Twelve samples were identified as the HPV6B3 variant, presenting the mutation (guanine) G474A (adenine), and one of them also showed the mutation (thymine) T369G. The other 13 patients were positive for HPV6B1 without nucleotide alterations. In the analysis of the HPV11 samples, all patients showed the mutations T137C and (cytosine) C380T. One patient also presented the nucleotide alteration T410C. None of the mutations found in the 32 analyzed samples resulted in amino acid changes. Patient age, local occurrence, and HIV infection did not show significant association with HPV infection. Besides, the data found in this study did not show a relationship with the geographical region of isolation when compared to other data from different regions of the world. In this way, despite the nucleotide alterations found, it was not possible to observe amino acid changes and variants grouping according to geographical region.

## 1. Introduction

Condyloma acuminata (CA), or genital warts, are benign proliferative epidermal or mucous lesions. Classical histopathological features of CA were recognized long ago and are characterized by acanthosis, papillomatosis, hyperkeratosis, parakeratosis, and koilocytosis [1]. These lesions, related to papillomavirus (PV) infections, are observed in animals, including cetaceans [2, 3], monkeys [4], and humans [5, 6]. In humans, CA is one of the most common sexually transmitted diseases; interestingly, gender bias has been associated with human papillomavirus 11 (HPV11) genital warts, whereby the proportion of HPV11 genital warts is three times higher in males than in females [7]. CA is caused by infection with human papillomavirus (HPV), mainly the low-risk types 6 and 11, though coinfections with high-risk HPV types can also occur [8–10].

Papillomaviruses are circular, double-stranded DNA viruses consisting of an icosahedral capsid 52-55 nm in diameter and with a genome length of 8 kb [11]. These viruses are classified in the* Papillomaviridae* family, which is characterized by large amounts of genetic diversity [12, 13]. There are currently more than 200 HPV types identified. The viruses can infect the squamous epithelium of the skin or the genital and oral mucosa [14–16]. Mucosal HPVs are classified according to their oncogenic potential: they could be high-risk, such as types 16 and 18, which are involved in oral, genital penile, anal and cervical carcinomas [17–21]; or low-risk, such as types 6 and 11, which are present in genital and anal warts and recurrent respiratory papillomatosis [22].

Former studies evaluating the genetic variation of the long control region (LCR) and E6 regions used standard reference genomes for comparative purposes [23–25]. These standard reference genomes for HPV6 are denoted as prototypic HPV6b, nonprototypic HPV6a and HPV6vc, and one standard reference genome for HPV11. However, a study based on phylogenetic analysis of complete genomes derived from published HPV6 and HPV11 variants proposed a new standard nomenclature for HPV6 and HPV11 [26]. Two deeply separate clades were observed for HPV6. The lineage A is formed by the reference genome HPV6b and the lineage B formed by the HPV6a, HPV6vc, and CAC301 sequences, corresponding to HPV6B3, HPV6B2, and HPV6B1 sublineages, respectively. The nomenclature proposed for the HPV11 lineage is based on two clades, referred to as sublineage A1, which includes variants clustering with the HPV11 reference genome, and sublineage A2, which includes all other variants.

Aggressiveness differences between HPVs 6 and 11 and between different cases of the same genotype could be related to intratypical genetics variants [27]. Several studies are trying to correlate these genetic alterations with biological and biochemical properties in an attempt to identify possible differences in the clinical-pathological characteristics of the disease [28].

Seedat et al. found duplications in the HPV6 LCR that could result in enhanced promoter activity. Thus, the duplication may cause an increase in the oncogenic potential of HPV6 variants ascribed to overexpression of E6 and E7 [29].

On the other hand, Flores-Díaz et al. did not observe an association of specific HPV11 variants with clinical disease. These could be explained by the higher conservation of HPV11 compared to HPV6 [30].

The analysis of HPV diversity is important for future vaccine strategies and to estimate the vaccine success in immunocompromised individuals [31].

HPV infection could be associated with some risk factors, such as high numbers of sexual partners, early age at start of sexual activity, tobacco smoking, number of pregnancies, alcohol, and previous sexually transmitted diseases (STDs) [32]. For example, tobacco, which contains nicotine, the main immunosuppressive constituent of cigarette smoke, has deleterious effects on systemic and local immunity, suppressing immune responses and increasing the susceptibility to HPV infection and persistence, leading to the development of HPV-associated lesions [33, 34]. The immune response has an important role in the elimination of many HPV infections. However, some infections cannot be eliminated and persist for many years, which is an additional risk factor for cancer development [35]. Individuals infected with ‎human immunodeficiency virus (HIV) are more susceptible to HPV infection. Deficiency in immune cells due to HIV infection results in instability of the immune system that should combat high- and low-risk HPVs, which facilitates virus persistence and lesion progression [36–38].

Therefore, our aims were to evaluate E6 early gene variability among HPV6 and HPV11 detected in CA samples obtained from a cohort of Brazilian patients and to correlate them with the clinical-pathologic data. We also conducted phylogenetic analysis to compare nucleotide sequences identified in our study with isolates previously described from other parts of the world.

## 2. Materials and Methods

### 2.1. Clinical Samples

Ethical permission was obtained from the Research Ethics Committee of the Institute of Biosciences, Letters and Exact Sciences of São Paulo State University, in the city of São José do Rio Preto, with the license number 1.529.236.

This study evaluated 25 samples positive for HPV6 and 7 samples positive for HPV11. The 32 samples were isolated from biopsies obtained from surgical sections of genital and perianal lesions from female patients attended at the Clinical Hospital of the University of São Paulo Medical School in Ribeirão Preto. The patient age ranged from 16 to 78 years, with a median age of 26 years. The local occurrence percentages of lesions in the analyzed patients were 50% in the genital region and 34.4% in the perianal region; 15.6% of samples showed no information (Table 1).

### 2.2. HPV Genotyping

The DNA from these samples was extracted using a phenol chloroform protocol [39], and the DNA integrity was evaluated via *β*-globin gene amplification, generating a 315-bp amplicon [40]. To amplify the human papillomavirus DNA present in these lesions, we used polymerase chain reaction (PCR) to target the L1 region of HPV. The reactions were processed in two amplification steps: in the first reaction, PGMy09 and PGMy11 oligonucleotides (Supplementary Table 1) were used to generate a 450-bp amplicon. The amplification mix consisted of 2.5 U of Taq DNA Polymerase (Sinapse Inc., Florida, USA), 2.5 *μ*l of 10X PCR Buffer, 5.6 *μ*M MgCl_2_, 0.2 mM dNTPs, 0.4 *μ*M of each primer, 500 ng of DNA, and nuclease free water, all of which added up to a final volume of 25.0 *μ*l. An initial denaturation step at 95°C for 9 min was conducted, followed by 40 cycles at 95°C for 1 min, 55°C for 1 min, and 72°C for 2 min and a final extension at 72°C for 5 min. In the nested PCR, GP5+ (5′-TTTGTTACTGTGGTAGATACTAC-3′) and GP6+ (5′-CTTATACTAAATGTCAAATAAAAA-3′) oligonucleotides were used to generate 150-bp amplification product. The amplification mix consisted of 2.5 U of Taq DNA Polymerase (Sinapse Inc., Florida, USA), 2.5 *μ*l of 10X PCR Buffer, 7.6 *μ*M magnesium chloride (MgCl_2)_, 0.064 mM deoxynucleotides (dNTPs), 0.48 *μ*M of each primer, 5.0 *μ*l of the product from the PGMy09 and PGMy11 reaction, and nuclease free water, all of which added up to a final volume of 25.0 *μ*l. An initial denaturation step at 95°C for 9 min was conducted, followed by 40 cycles at 94°C for 30 seconds, 45°C for 30 seconds and 72°C for 30 seconds, and a final extension at 72°C for 8 min. Purified products were sequenced via the Sanger method using the primers PGMy09/11 and GP5+/6+ [41].

### 2.3. E6 Amplification and Cloning

For nucleotide variability analysis, the complete E6 gene sequences of HPV6- and HPV11-positive samples were amplified using PCR with specific primers (HPV6: F: 5′ GGGGGATCCGAATTCATGGAAAGTGCAAATGC 3′ and R: 5′ GGAAGACATGTTACCCTAGGATCCAAGCTTCAC 3′; HPV11: F: 5′ AAAATTAGCAGACGAGGCATT 3′ and R: 5′ AGATGAGGTGGACAAGGTGG 3′) [42]. The amplification mix consisted of 6.0 U of a proofreading polymerase (High Fidelity Enzyme Mix, Fermentas, Vilnius, Lithuania), 5.0 *μ*l of 10X High Fidelity PCR Buffer, 1.5 mM MgCl_2_, 0.24 mM dNTPs, 0.4 *μ*M of each primer, 500 ng of DNA, and nuclease free water, all of which added up to a final volume of 50.0 *μ*l. An initial denaturation step at 95°C for 5 min was conducted, followed by 35 cycles at 95°C for 1 min, 55°C for 1 min, and 72°C for 2 min and a final extension at 72°C for 8 min. The amplification products were 467 bp for HPV6 E6 and 569 bp for HPV11 E6.

To obtain more reliable sequencing data, the amplified E6 region from each patient was ligated to the pJET1.2/blunt cloning vector of the Clone JET PCR Cloning Kit (Thermo Scientific, Massachusetts, USA) following the manufacturer's instructions. The cloned products were transformed [43] into chemically competent DH5*α* Z* Escherichia coli* (Zymo Research, California, USA) via the thermal shock method. After transformation, bacteria were spread onto Petri plates containing solid Luria-Bertani (LB) medium and 0.1 mg/mL of ampicillin. Samples were incubated at 37°C for 16 hours. Subsequently, five colonies of each sample were selected and incubated in liquid LB medium with 0.1 mg/mL of ampicillin at 37°C for 16 hours and shaking at 250 rpm. After bacterial growth, the plasmid DNA was extracted using the GeneJET™ Plasmid Miniprep Kit (Fermentas, Vilnius, Lithuania) following the manufacturer's instructions.

### 2.4. Nucleotide Alteration Detection

Purified products were sequenced via the Sanger method using cloning vector primers (pJET1.2 Forward Sequencing Primer: 5′ CGACTCACTATAGGGAGAGCGGC 3′ and pJET1.2 Reverse Sequencing Primer: 5′ CTGCCATGGAAAATCGATGTTCTT 3′).

### 2.5. Datasets and Sequence Analysis

Sequence quality was evaluated using the Electropherogram Quality Analysis program, available online at <http://asparagin.cenargen.embrapa.br/phph/>. Comparisons between the sequences acquired and those previously added to GenBank were conducted using BLAST (Basic Local Alignment Search Tool, available at <http://www.ncbi.nlm.nih.gov/BLAST>).

All sequences were edited using the BioEdit 7.0.9.0 package to remove vector fragments and to analyze solely the complete sequence of the E6 gene. The alignment between the prototype sequence HPV6A (accession number: X00203), nonprototype sequences HPV6B1 (accession number: AF092932), HPV6B2 (accession number: FM875941), and HPV6B3 (accession number: L41216), HPV11A1 reference sequence (accession number: M14119) and HPV11A2 (accession number: FN870447), and the sequences obtained in this study was performed using the CLUSTAL W software nested in the BioEdit 7.0.9.0 package [44, 45].

The sequences generated in this study were submitted to GenBank; the accession numbers are listed in Supplementary Table 2.

To perform the phylogenetic analysis, datasets were assembled, including the nucleotide sequences generated in this study, the reference sequences, and other sequences available on GenBank for each HPV type. The HPV6 and HPV11 datasets consist of 184 and 101 nucleotide sequences, respectively, both with 453 residues. The GenBank accession numbers of all of the sequences are presented in Supplementary Table 3.

### 2.6. Phylogenetic Analysis

Phylogenetic trees were reconstructed with the Maximum Likelihood method using the* PhyML *program through the ATGC platform from the South of France Bioinformatics Laboratories (http://www.atgc-montpellier.fr/phyml/) [46]. The substitution models were calculated for both HPV types (6 and 11) using* jModel Test *software [47]. Bootstrapping of 1000 replicates was used to calculate branch support. Values over 70% were considered significant.

### 2.7. Statistical Analysis

The Kruskal-Wallis test was used to determine if there were significant associations between HPV presence and HIV viral load and between HPV presence and patient age. The HIV viral load of the samples was determined by the branched-chain DNA assay (Versant HIV RNA test, Version 3.0, lower limit of quantification 50 copies/ml; Siemens Healthcare, Erlangen, Germany) and values above 50 HIV-1 RNA copies/ml were considered HIV-positive.* p*-values <0.05 were considered as statistically significant.

The Pearson Chi-Square, Likelihood Ratio Chi-Square, and Fisher's exact tests were employed to identify the association of HPV infection with the risk factor (HIV presence). These tests were also used to analyze the associations between nucleotide alterations and the anatomical location of the lesions.* p*-values <0.05 were considered as statistically significant.

## 3. Results

### 3.1. Clinical Characteristics

Regarding the age factor, 61.3% of patients were between 16 and 29 years old, and 38.7% were more than 30 years old. However, neither patient age nor local occurrence was significantly associated with HPV6 or HPV11 presence. Related to the risk factors, HIV was detected in 21.9% of patients, and statistical analysis did not show significant association between HPV and HPV coinfection. It was not possible to perform statistical analysis on HPV infection, alcohol consumption, and tobacco smoking due to missing patient data.

### 3.2. Nucleotide Alteration Detection

#### 3.2.1. HPV6

After sequence analysis of the 25 HPV6 samples, it was noted that 12 (48%) samples belong to the HPV6B3 (L42216) variant and that all of these samples presented the G474A mutation compared to the prototype E6 sequence. Moreover, sample BR_CA06_B3 showed one more nucleotide alteration in position 369 of the genome, consisting of a change from T to G (Table 2). The other 13 (52%) HPV6 samples belong to the HPV6B1 (AF092932) variant and did not show additional nucleotide alterations.

Modeltest was performed to determine the best substitution model for phylogenetic reconstruction. The substitution model selected for the HPV6 dataset was Tamura-Nei + I + G (TrN + I +G). A maximum likelihood phylogenetic tree was reconstructed using PhyML based on the selected model with a bootstrap of 1000 replicates.

**Figure 1:**
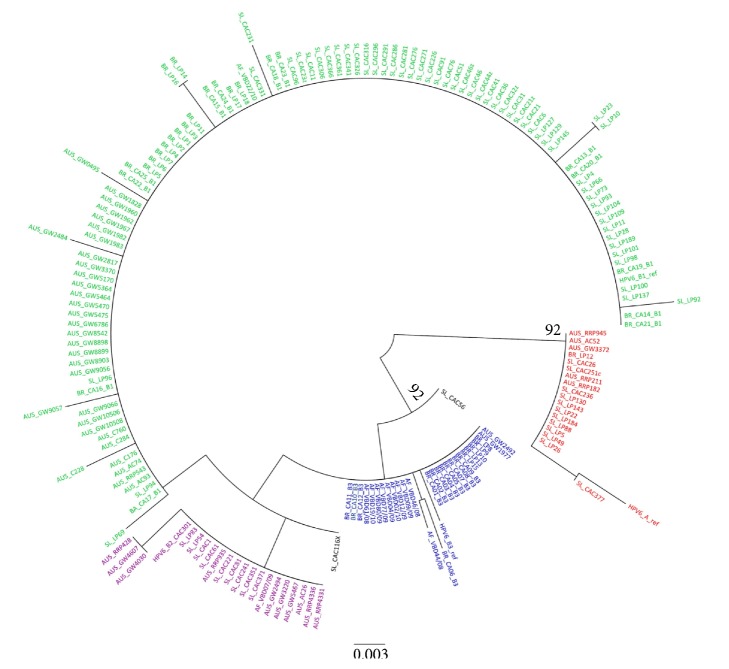
Unrooted maximum likelihood phylogenetic tree for HPV6 variants reconstructed based on a dataset of 184 nucleotide sequences of 453 residues with the TrN + I +G substitution model and a bootstrap of 1000 replicates. Values above 70% were considered significant. (African variants: AF; Australian variants: AUS; Brazilian variants: BR; Slovenian variants: SL.) Red: HPV6A; green: HPV6B1; purple: HPV6B2; blue: HPV6B3. Sequences in black are HPV6B that cannot be classified into sublineages.

The phylogenetic tree obtained from the analysis was split into two main branches, with strong branch support (Figure 1). One branch, with a bootstrap of 92, grouped the HPV6A prototype sequence with the isolates related to this variant. The other branch, also with a bootstrap of 92, grouped all HPV6B variants and was divided into two secondary branches. One secondary branch included the HPV6B3 sequences, including 12 sequences from this study together with the reference sequence. The second branch included the HPV6B1 and HPV6B2 sublineages. The 13 sequences from this study grouped in a monophyletic branch, with no branch support, with the HPV6B1 sublineage reference sequence and related sequences available in the literature. Thus, the sequences did not group according to either geographical regions or the anatomical site of infection.

#### 3.2.2. HPV11

Analysis of seven HPV11 samples revealed that all samples belong to the HPV11A2 variant but only the sample BR_CA28_A2 showed the nucleotide alteration T410C (Table 3) compared to the sequence LP19 (accession number: FN870447) classified as HPV11A2 by Burk et al., 2011 [26]. None of the mutations in the 32 analyzed samples resulted in amino acid changes.

Modeltest was used to determine the best substitution model for phylogenetic reconstruction, and the HKY model was selected. A maximum likelihood phylogenetic tree was reconstructed using PhyML based on the selected model with a bootstrap of 1000 replicates.

**Figure 2:**
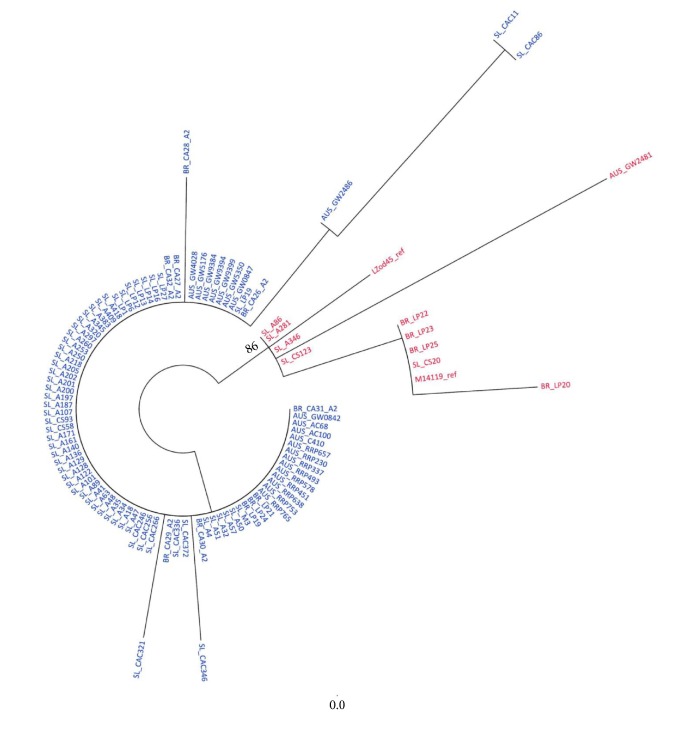
Unrooted maximum likelihood phylogenetic tree for HPV11 reconstructed based on a dataset of 101 nucleotide sequences of 453 residues with the TrN + I +G substitution model and a bootstrap of 1000 replicates. Values above 70% were considered significant. (Australian variants: AUS; Brazilian variants: BR; Slovenian variants: SL.) Red: HPV11A1; blue: HPV11A2.

The phylogenetic tree segregated into two main branches. One of them, with a bootstrap of 86, contained the HPV11A1 prototype sequence (M14119), the LZod45 variant, and isolates from other studies (Figure 2). The second branch grouped the HPV11A2 sequences from this study and the Brazilian, Slovenian, and Australian isolates. Thus, it was observed that sequences did not group according to either the geographical region from which they were isolated or the anatomical site of detection.

## 4. Discussion

The HPV11 E6 and E7 proteins play important roles in ensuring a productive viral life cycle, facilitating episomal maintenance of the viral genome [48]. Mutations in these regions may cause differences in the infection potential of the virus [31], possibly due to the different interactions of the virus with host cellular mechanisms that may modulate its clinical course [31].

In this study, 12 CA samples belong to the HPV6B3 variant, and all of them had a nucleotide alteration in position 474 of the HPV genome. This mutation was also found in Australian anogenital samples [49] and in Brazilian recurrent respiratory papillomatosis samples [42]. In contrast, the nucleotide alteration at position 369 in the virus genome observed in our study was not observed in both studies. Despite the nucleotide substitutions detected in the E6 sequence, amino acid changes were not observed in our study. Although the differences between the variants have been small, the HPV6B1 variant was slightly more frequent in this study. These data corroborate the report that HPV6B1 is the most common variant in recurrent respiratory papillomatosis and genital warts [23, 42, 49]. This variant was highly conserved in genital lesions analyzed in this study since it did not show any nucleotide alteration. One CA sample from this study that was HPV11-positive showed alteration in relation to the HPV11A2 sequence (accession number: FN FN870447). Nucleotide alteration at position 410 of the virus genome was found in recurrent respiratory papillomatosis [42]. However, this alteration did not result in amino acid changes. The presence of HPV11 variants instead of prototype sequences has also been observed in other studies [23, 42]. Different sublineages of the same HPV genotype could result in alterations in viral infection persistence and the progression of precursor lesions and could also affect viral assembly, the immune response, pathogenicity, and p53 degradation [50].

In the phylogenetic analysis, it was not possible to observe the genomic variant distribution according to the geographical region from which the virus was isolated, as observed with HPVs 16 and 18. These two types of HPV originated and speciated in Africa and then spread and diversified through human migrations. Thus, variants are different in several geographical locations [51, 52]. In contrast to genotypes 16 and 18, previous studies and this study suggest that the genomic diversities of HPV6 and 11 isolated from several anatomical sites are not correlated to the place from which sample was isolated [23, 42, 48, 53]. We suggest that there is no association between the anatomical site of lesions and the HPV6 and HPV11 variants. Unlike this, Jelen et al. [54] observed an association of sublineages B1 and B3 with anogenital infections. The relation between anogenital lesions and sublineage B1 was also found by Danielewski et al. [49].

More studies will be necessary to analyze the influence of HPV intratypic genetic variants on the increased risk of carcinogenic development [55]. Data observed in the present study and in the literature indicate that types and variants of papillomavirus identification and risk factor correlations could be useful for risk analysis and lesion management strategies.

Risk factors for HPV infections could include genital contact, early age at the start of sexual activity, number of lifetime sexual partners, previous sexually transmitted diseases, tobacco smoking, and alcohol consumption [34]. Evasion of the immune response to HPV is critical for a successful infection [35]. Women infected with HIV have a higher risk to facilitate HPV persistence and a reduced capacity to control the oncogenic viral processes [56]. However, in the present study we did not observe a significant association of HPV and HIV coinfection corroborating a study conducted in Africa with men whereupon low-risk HPV was not associated with HIV incidence [57]. Regarding the age factor, 61.3% of the patients were between 16 and 29 years old; the other patients were more than 30 years old. The lower occurrence of HPV in older women could be due to infection elimination and natural immunity. However, this decrease could also be related to the increase in safe sexual behavior among older women [58]. The statistical analysis in this study revealed no association between HPV persistence and patient age, a result that was also observed in cervical samples [59].

## 5. Conclusion

In the present study, it was possible to observe 12 HPV6B3 variants, all of which presented the G474A mutation. One HPV6B3 sample also showed the T369G mutation. The other HPV6 samples were determined to be HPV6B1 variants and did not present nucleotide alterations. T410C alteration was found in one of the HPV11 samples. Phylogenetic analysis showed no association between geographical or anatomical site of HPV detection and HPV6 or HPV11 variants.

## Figures and Tables

**Table 1:** Characterization of the 32 samples regarding to the factors age, lesion, HIV coinfection, alcohol consumption, and tobacco smoking.

Sample	Age (Years)	Condyloma	HPV type	HIV	HIV viral load (HIV-1 RNA copies/ml)	Alcohol consumption	Tobacco smoking

BR_CA26_A2	51	-	HPV11	Neg	< 50	-	No
BR_CA13_B1	26	Perianal	HPV6	Neg	< 50	No	No
BR_CA01_B3	42	-	HPV6	Neg	< 50	No	Yes
BR_CA27_A2	49	Genital	HP11	Pos	11543	Yes	Yes
BR_CA02_B3	22	Genital	HPV6	Neg	< 50	-	No
BR_CA03_B3	50	Perianal	HPV6	Neg	< 50	No	Yes
BR_CA14_B1	20	Perianal	HPV6	Neg	< 50	Yes	No
BR_CA28_A2	39	Genital	HPV11	Neg	< 50	No	No
BR_CA15_B1	37	Perianal	HPV6	Neg	< 50	No	No
BR_CA16_B1	24	-	HPV6	Neg	< 50	-	-
BR_CA29_A2	26	Genital	HPV11	Pos	18656	Yes	Yes
BR_CA04_B3	42	Genital	HPV6	Neg	< 50	No	No
BA_CA17_B1	78	-	HPV6	Neg	< 50	No	No
BR_CA05_B3	21	Genital	HPV6	Neg	< 50	No	No
BR_CA18_B1	21	Genital	HPV6	Neg	< 50	No	No
BR_CA06_B3	45	Genital	HPV6	Pos	20657	No	Yes
BR_CA30_A2	29	Genital	HPV11	Pos	25678	-	Yes
BR_CA32_A2	29	Genital	HPV11	Pos	22980	-	Yes
BR_CA07_B3	-	-	HPV6	Pos	15000	-	-
BR_CA08_B3	39	Perianal	HPV6	Pos	35678	-	-
BR_CA31_A2	22	Perianal	HPV11	Neg	< 50	-	
BR_CA19_B1	16	Genital	HPV6	Neg	< 50	No	No
BR_CA09_B3	23	Genital	HPV6	Neg	< 50	No	Yes
BR_CA10_B3	21	Genital	HPV6	Neg	< 50	-	-
BR_CA20_B1	53	Perianal	HPV6	Neg	< 50	-	Yes
BR_CA11_B3	24	Perianal	HPV6	Neg	< 50	-	-
BR_CA12_B3	21	Genital	HPV6	Neg	< 50	No	No
BR_C21_B1	33	Genital	HPV6	Neg	< 50		-
BR_C22_B1	19	Genital	HPV6	Neg	< 50	No	No
BR_C23_B1	19	Perianal	HPV6	Neg	< 50	No	No
BR_C24_B1	27	Perianal	HPV6	Neg	< 50	-	-
BR_C25_B1	17	Perianal	HPV6	Neg	< 50	No	No

subtitle: -: lack of information; Neg: negative; Pos: positive.

**Table 2:** Nucleotide alterations in the E6 genes of HPV6 isolates. Genomic positions are indicated in the upper part of the table, and the mutations are vertically indicated. Conserved nucleotides in relation to the reference sequence are shown in gray. The column “Number of samples” indicates the number of patients in which the isolates are identical to the specified variant.

	HPV6 samples	Nucleotide positions		
		3	4	Number of samples
		6	7	
		9	4	

B3	B3_ref	T	G	
	BR_CA01, BR_CA02, BR_CA03, BR_CA04, BR_CA05, BR_CA07, BR_CA08, BR_CA09, BR_CA10, BR_CA11, BR_CA12	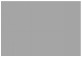	A	11
	BR_CA06	G	A	1

B1	B1_ref	T	A	
	BR_CA13, BR_CA14, BR_CA15, BR_CA16, BR_CA17, BR_CA18, BR_CA19, BR_CA20, BR_C21, BR_C22, BR_C23, BR_C24, BR_C25	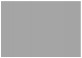	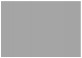	13

**Table 3:** Nucleotide alterations in the E6 genes of HPV11 isolates. Genomic positions are indicated in the upper part of the table, and the mutation is horizontally indicated. The column “Number of samples” indicates the number of patients in which the isolates are identical to the specified variant.

HPV 11 sample	Nucleotide sample	
	410	Number of samples

A2 (LP19-FN 870447)	T	
BR_CA28	C	1

## Data Availability

“Sequence” data that support the findings of this study have been deposited in GenBank with the accession codes “MF375424”, “MF375425”, “MF375426”, “MF375427”, “MF375428”, “MF375429”, “MF375430”, “MF375431”, “MF375432”, “MF375433”, “MF375434”, “MF375435”, “MF375436”, “MF375437”, “MF375438”, “MF375439”, “MF375440”, “MF375441”, “MF375442”, “MF375443”, “MF375444”, “MF375445”, “MF375446”, “MF375447”, “MF375448”, “MF375449”, “MF375450”, “MF375451”, “MF375452”, “MF375453”, “MF375454”, and “MF375455”.

## Abbreviations

G: Guanine
A: Adenine
T: Thymine
C: Cytosine
CA: Condyloma acuminata
HPV: Human papillomavirus
HIV: Human immunodeficiency virus
PV: Papillomavirus
STD: Sexually transmitted disease
PCR: Polymerase chain reaction
MgCl_2_: Magnesium chloride
dNTPs: Deoxynucleotide
LB: Luria-Bertani
BLAST: Basic local alignment search tool.

## Supplementary Materials

Supplementary 1 Table 1: oligonucleotides sequences that comprise PGMy09 and PGMy11.

Supplementary 2 Table 2: sequences generated on the study and their accession numbers on GenBank.

Supplementary 3 Table 3: HPV6 and HPV11 sequences available on GenBank used on the phylogenetic analysis [42, 49, 60–67].
