# Supplementary material for: Analysis of Nucleotide Alterations in the E6 Genomic Region of Human Papillomavirus Types 6 and 11 in Condyloma Acuminatum Samples from Brazil
Source: Adv Virol. 2019 May 2;2019:5697573. doi: 10.1155/2019/5697573 (PMC6521423; doi:10.1155/2019/5697573)
Supplement: Supplementary 1 — Table 1: oligonucleotides sequences that comprise PGMy09 and PGMy11. [file 5697573.f1.doc]

Supplementary file 1

Table 1. Oligonucleotides sequences that comprise PGMy09 and PGMy11.

| **Oligonucleotide** | **Sequence** |
| --- | --- |
| PGMY11-A | 5’ GCA CAG GGA CAT AAC AAT GG 3’ |
| PGMY11-B | 5’ GCG CAG GGC CAC AAT AAT GG 3’ |
| PGMY11-C | 5’ GCA CAG GGA CAT AAT AAT GG 3’ |
| PGMY11-D | 5’ GCC CAG GGC CAC AAC AAT GG 3’ |
| PGMY11-E | 5’ GCT CAG GGT TTA AAC AAT GG 3’ |
| PGMY09-F | 5’ CGT CCC AAA GGA AAC TGA TC 3’ |
| PGMY09-G | 5’ CGA CCT AAA GGA AAC TGA TC 3’ |
| PGMY09-H | 5’ CGT CCA AAA GGA AAC TGA TC 3’ |
| PGMY09-I* | 5’ G CCA AGG GGA AAC TGA TC 3’ |
| PGMY09-J | 5’ CGT CCC AAA GGA TAC TGA TC 3’ |
| PGMY09-K | 5’ CGT CCA AGG GGA TAC TGA TC 3’ |
| PGMY09-L | 5’ CGA CCT AAA GGG AAT TGA TC 3’ |
| PGMY09-M | 5’ CGA CCT AGT GGA AAT TGA TC 3’ |
| PGMY09-N | 5’ CGA CCA AGG GGA TAT TGA TC 3’ |
| PGMY09-P* | 5’ G CCC AAC GGA AAC TGA TC 3’ |
| PGMY09-Q | 5’ CGA CCC AAG GGA AAC TGG TC 3’ |
| PGMY09-R | 5’ CGT CCT AAA GGA AAC TGG TC 3’ |
| HMBO1 | 5’ GCG ACC CAA TGC AAA TTG GT 3’ |
