# Supplementary material for: Analysis of Nucleotide Alterations in the E6 Genomic Region of Human Papillomavirus Types 6 and 11 in Condyloma Acuminatum Samples from Brazil
Source: Adv Virol. 2019 May 2;2019:5697573. doi: 10.1155/2019/5697573 (PMC6521423; doi:10.1155/2019/5697573)
Supplement: Supplementary 2 — Table 2: sequences generated on the study and their accession numbers on GenBank. [file 5697573.f2.docx]

**Supplementary file 2**

**Table 2.** Sequences generated on the study and their accession numbers on GenBank.

| **Sample** | **Accession number** |
| --- | --- |
| BR_CA01_B3 | MF375424 |
| BR_CA02_B3 | MF375425 |
| BR_CA03_B3 | MF375426 |
| BR_CA04_B3 | MF375427 |
| BR_CA05_B3 | MF375428 |
| BR_CA06_B3 | MF375429 |
| BR_CA07_B3 | MF375430 |
| BR_CA08_B3 | MF375431 |
| BR_CA09_B3 | MF375432 |
| BR_CA10_B3 | MF375433 |
| BR_CA11_B3 | MF375434 |
| BR_CA12_B3 | MF375435 |
| BR_CA13_B1 | MF375436 |
| BR_CA14_B1 | MF375437 |
| BR_CA15_B1 | MF375438 |
| BR_CA16_B1 | MF375439 |
| BR_CA17_B1 | MF375440 |
| BR_CA18_B1 | MF375441 |
| BR_CA10_B1 | MF375442 |
| BR_CA20_B1 | MF375443 |
| BR_CA21_B1 | MF375444 |
| BR_CA22_B1 | MF375445 |
| BR_CA23_B1 | MF375446 |
| BR_CA24_B1 | MF375447 |
| BR_CA25_B1 | MF375448 |
| BR_CA26_A2 | MF375449 |
| BR_CA27_A2 | MF375450 |
| BR_CA28_A2 | MF375451 |
| BR_CA29_A2 | MF375452 |
| BR_CA30_A2 | MF375453 |
| BR_CA31_A2 | MF375454 |
| BR_CA32_A2 | MF375455 |
