# Supplementary material for: Analysis of Nucleotide Alterations in the E6 Genomic Region of Human Papillomavirus Types 6 and 11 in Condyloma Acuminatum Samples from Brazil
Source: Adv Virol. 2019 May 2;2019:5697573. doi: 10.1155/2019/5697573 (PMC6521423; doi:10.1155/2019/5697573)
Supplement: Supplementary 3 — Table 3: HPV6 and HPV11 sequences available on GenBank used on the phylogenetic analysis [42, 49, 60–67]. [file 5697573.f3.docx]

**Supplementary file 3**

**Table 3.** HPV6 and HPV11 sequences available on GenBank used on the phylogenetic analysis ([Combrinck et al., 2012](#_ENREF_1); [Danielewski et al., 2013](#_ENREF_2); [Dartmann et al., 1986](#_ENREF_3); [de Matos et al., 2013](#_ENREF_4); [Hofmann et al., 1995](#_ENREF_5); [Kocjan et al., 2011](#_ENREF_6); [Kovelman et al., 1999](#_ENREF_7); [Maver et al., 2011](#_ENREF_8); [Schwarz et al., 1983](#_ENREF_9); [Wu et al., 2009](#_ENREF_10))

| **Sample** | **Accession number** | **Reference** |
| --- | --- | --- |
| HPV6_B3_ref | L41216.1 | HOFMANN et al., 1995 |
| HPV6_A_ref | X00203.1 | SCHWARZ et al., 1983 |
| HPV6_B1_ref | AF092932.1 | KOVELMAN et al., 1999 |
| HPV6_B2_CAC301 | FR751328.1 | KOCJAN et al., 2009 |
| SL_LP96 | FM897037.1 | KOCJAN et al., 2009 |
| SL_LP94 | FM897036.1 | KOCJAN et al., 2009 |
| SL_LP100 | FM897035.1 | KOCJAN et al., 2009 |
| SL_LP69 | FM897034.1 | KOCJAN et al., 2009 |
| SL_LP98 | FM897033.1 | KOCJAN et al., 2009 |
| SL_LP101 | FM897032.1 | KOCJAN et al., 2009 |
| SL_LP189 | FM897031.1 | KOCJAN et al., 2009 |
| SL_LP28 | FM897030.1 | KOCJAN et al., 2009 |
| SL_LP11 | FM897029.1 | KOCJAN et al., 2009 |
| SL_LP109 | FM897028.1 | KOCJAN et al., 2009 |
| SL_LP104 | FM897027.1 | KOCJAN et al., 2009 |
| SL_LP93 | FM897026.1 | KOCJAN et al., 2009 |
| SL_LP73 | FM897025.1 | KOCJAN et al., 2009 |
| SL_LP66 | FM897024.1 | KOCJAN et al., 2009 |
| SL_LP4 | FM897023.1 | KOCJAN et al., 2009 |
| SL_LP23 | FM897022.1 | KOCJAN et al., 2009 |
| SL_LP10 | FM897021.1 | KOCJAN et al., 2009 |
| SL_LP92 | FM897020.1 | KOCJAN et al., 2009 |
| SL_LP83 | FM897019.1 | KOCJAN et al., 2009 |
| SL_LP54 | FM897018.1 | KOCJAN et al., 2009 |
| SL_LP49 | FM897017.1 | KOCJAN et al., 2009 |
| SL_LP26 | FM897016.1 | KOCJAN et al., 2009 |
| SL_LP5 | FM897015.1 | KOCJAN et al., 2009 |
| SL_LP88 | FM897014.1 | KOCJAN et al., 2009 |
| SL_LP184 | FM897013.1 | KOCJAN et al., 2009 |
| SL_LP22 | FM897012.1 | KOCJAN et al., 2009 |
| SL_LP145 | FM897011.1 | KOCJAN et al., 2009 |
| SL_LP143 | FM897010.1 | KOCJAN et al., 2009 |
| SL_LP137 | FM897009.1 | KOCJAN et al., 2009 |
| SL_LP130 | FM897008.1 | KOCJAN et al., 2009 |
| SL_LP129 | FM897007.1 | KOCJAN et al., 2009 |
| SL_LP127 | FM897006.1 | KOCJAN et al., 2009 |
| SL_CAC1 | FM875945.1 | KOCJAN et al., 2009 |
| SL_CAC6 | FM875968.1 | KOCJAN et al., 2009 |
| SL_CAC21 | FM875950.1 | KOCJAN et al., 2009 |
| SL_CAC21z | FM875983.1 | KOCJAN et al., 2009 |
| SL_CAC31 | FM875969.1 | KOCJAN et al., 2009 |
| SL_CAC32z | FM875982.1 | KOCJAN et al., 2009 |
| SL_CAC36 | FM875953.1 | KOCJAN et al., 2009 |
| SL_CAC41 | FM875954.1 | KOCJAN et al., 2009 |
| SL_CAC44z | FM875984.1 | KOCJAN et al., 2009 |
| SL_CAC46 | FM875973.1 | KOCJAN et al., 2009 |
| SL_CAC46z | FM875985.1 | KOCJAN et al., 2009 |
| SL_CAC51 | FM875951.1 | KOCJAN et al., 2009 |
| SL_CAC61 | FM875946.1 | KOCJAN et al., 2009 |
| SL_CAC76 | FM875955.1 | KOCJAN et al., 2009 |
| SL_CAC81 | FM875947.1 | KOCJAN et al., 2009 |
| SL_CAC91 | FM875967.1 | KOCJAN et al., 2009 |
| SL_CAC116X | FM875965.1 | KOCJAN et al., 2009 |
| SL_CAC221 | FM875948.1 | KOCJAN et al., 2009 |
| SL_CAC226 | FM875952.1 | KOCJAN et al., 2009 |
| SL_CAC236 | FM875979.1 | KOCJAN et al., 2009 |
| SL_CAC241 | FM875942.1 | KOCJAN et al., 2009 |
| SL_CAC271 | FM875970.1 | KOCJAN et al., 2009 |
| SL_CAC276 | FM875972.1 | KOCJAN et al., 2009 |
| SL_CAC281 | FM875956.1 | KOCJAN et al., 2009 |
| SL_CAC286 | FM875957.1 | KOCJAN et al., 2009 |
| SL_CAC291 | FM875958.1 | KOCJAN et al., 2009 |
| SL_CAC296 | FM875959.1 | KOCJAN et al., 2009 |
| SL_CAC316 | FM875960.1 | KOCJAN et al., 2009 |
| SL_CAC326 | FM875961.1 | KOCJAN et al., 2009 |
| SL_CAC341 | FM875962.1 | KOCJAN et al., 2009 |
| SL_CAC351 | FM875943.1 | KOCJAN et al., 2009 |
| SL_CAC361 | FM875963.1 | KOCJAN et al., 2009 |
| SL_CAC366-1 | FM875971.1 | KOCJAN et al., 2009 |
| SL_CAC371 | FM875944.1 | KOCJAN et al., 2009 |
| SL_CAC306 | FR751337.1 | KOCJAN et al., 2009 |
| SL_CAC11 | FR751334.1 | KOCJAN et al., 2009 |
| SL_CAC23z | FR751330.1 | KOCJAN et al., 2009 |
| SL_CAC26 | FR751322.1 | KOCJAN et al., 2009 |
| SL_CAC56 | FR751326.1 | KOCJAN et al., 2009 |
| SL_CAC96 | FR751335.1 | KOCJAN et al., 2009 |
| SL_CAC231 | FR751336.1 | KOCJAN et al., 2009 |
| SL_CAC251c | FR751321.1 | KOCJAN et al., 2009 |
| SL_CAC331 | FR751327.1 | KOCJAN et al., 2009 |
| SL_CAC377 | FR751325.1 | KOCJAN et al., 2009 |
| AF_VBD61/08 | JN573174.1 | COMBRINCK et al., 2011 |
| AF_VBD22/10 | JN573173.1 | COMBRINCK et al., 2011 |
| AF_VBD19/10 | JN573172.1 | COMBRINCK et al., 2011 |
| AF_VBD07/09 | JN573171.1 | COMBRINCK et al., 2011 |
| AF_VBD80/09 | JN573170.1 | COMBRINCK et al., 2011 |
| AF_VBD77/09 | JN573169.1 | COMBRINCK et al., 2011 |
| AF_VBD04/09 | JN573168.1 | COMBRINCK et al., 2011 |
| AF_VBD02/10 | JN573167.1 | COMBRINCK et al., 2011 |
| AF_VBD12/09 | JN573166.1 | COMBRINCK et al., 2011 |
| AF_VBD09/09 | JN573165.1 | COMBRINCK et al., 2011 |
| AF_VBD46/08 | JN573164.1 | COMBRINCK et al., 2011 |
| AF_VBD44/08 | JN573163.1 | COMBRINCK et al., 2011 |
| BR_LP18 | KC285855.1 | DE MATOS et al., 2013 |
| BR_LP17 | KC285854.1 | DE MATOS et al., 2013 |
| BR_LP16 | KC285853.1 | DE MATOS et al., 2013 |
| BR_LP15 | KC285852.1 | DE MATOS et al., 2013 |
| BR_LP14 | KC285851.1 | DE MATOS et al., 2013 |
| BR_LP13 | KC285850.1 | DE MATOS et al., 2013 |
| BR_LP12 | KC285849.1 | DE MATOS et al., 2013 |
| BR_LP11 | KC285848.1 | DE MATOS et al., 2013 |
| BR_LP3 | KC285847.1 | DE MATOS et al., 2013 |
| BR_LP1 | KC285846.1 | DE MATOS et al., 2013 |
| BR_LP2 | KC285845.1 | DE MATOS et al., 2013 |
| BR_LP4 | KC285844.1 | DE MATOS et al., 2013 |
| BR_LP10 | KC285843.1 | DE MATOS et al., 2013 |
| BR_LP9 | KC285842.1 | DE MATOS et al., 2013 |
| BR_LP8 | KC285841.1 | DE MATOS et al., 2013 |
| BR_LP7 | KC285840.1 | DE MATOS et al., 2013 |
| BR_LP6 | KC285839.1 | DE MATOS et al., 2013 |
| BR_LP5 | KC285838.1 | DE MATOS et al., 2013 |
| AUS_GW0495F | KC300156.1 | DANIELEWSKI et al., 2013 |
| AUS_GW1828M | KC300159.1 | DANIELEWSKI et al., 2013 |
| AUS_GW1960B | KC300160.1 | DANIELEWSKI et al., 2013 |
| AUS_GW1962F | KC300161.1 | DANIELEWSKI et al., 2013 |
| AUS_GW1967E | KC300162.1 | DANIELEWSKI et al., 2013 |
| AUS_GW1977M | KC300163.1 | DANIELEWSKI et al., 2013 |
| AUS_GW1982Y | KC300164.1 | DANIELEWSKI et al., 2013 |
| AUS_GW1983M | KC300165.1 | DANIELEWSKI et al., 2013 |
| AUS_GW2484W | KC300166.1 | DANIELEWSKI et al., 2013 |
| AUS_GW2492A | KC300167.1 | DANIELEWSKI et al., 2013 |
| AUS_GW2494E | KC300168.1 | DANIELEWSKI et al., 2013 |
| AUS_GW2817WA | KC300169.1 | DANIELEWSKI et al., 2013 |
| AUS_GW3220E | KC300170.1 | DANIELEWSKI et al., 2013 |
| AUS_GW3370M | KC300171.1 | DANIELEWSKI et al., 2013 |
| AUS_GW3372R | KC300172.1 | DANIELEWSKI et al., 2013 |
| AUS_GW4030F | KC300173.1 | DANIELEWSKI et al., 2013 |
| AUS_GW4607M | KC300174.1 | DANIELEWSKI et al., 2013 |
| AUS_GW5170R | KC300175.1 | DANIELEWSKI et al., 2013 |
| AUS_GW5364D | KC300176.1 | DANIELEWSKI et al., 2013 |
| AUS_GW5464R | KC300177.1 | DANIELEWSKI et al., 2013 |
| AUS_GW5467L | KC300178.1 | DANIELEWSKI et al., 2013 |
| AUS_GW5470R | KC300179.1 | DANIELEWSKI et al., 2013 |
| AUS_GW5475P | KC300180.1 | DANIELEWSKI et al., 2013 |
| AUS_GW6786B | KC300188.1 | DANIELEWSKI et al., 2013 |
| AUS_GW8542H | KC300181.1 | DANIELEWSKI et al., 2013 |
| AUS_GW8898T1 | KC300182.1 | DANIELEWSKI et al., 2013 |
| AUS_GW8899J | KC300183.1 | DANIELEWSKI et al., 2013 |
| AUS_GW8903J | KC300184.1 | DANIELEWSKI et al., 2013 |
| AUS_GW9056T | KC300185.1 | DANIELEWSKI et al., 2013 |
| AUS_GW9057J | KC300186.1 | DANIELEWSKI et al., 2013 |
| AUS_GW9066D | KC300187.1 | DANIELEWSKI et al., 2013 |
| AUS_GW10506J | KC300157.1 | DANIELEWSKI et al., 2013 |
| AUS_GW10508M | KC300158.1 | DANIELEWSKI et al., 2013 |
| AUS_C760 | KC300155.1 | DANIELEWSKI et al., 2013 |
| AUS_C284 | KC300154.1 | DANIELEWSKI et al., 2013 |
| AUS_C228 | KC300153.1 | DANIELEWSKI et al., 2013 |
| AUS_C176 | KC300152.1 | DANIELEWSKI et al., 2013 |
| AUS_AC74 | KC300151.1 | DANIELEWSKI et al., 2013 |
| AUS_AC52 | KC300150.1 | DANIELEWSKI et al., 2013 |
| AUS_AC26 | KC300149.1 | DANIELEWSKI et al., 2013 |
| AUS_RRP43364A | KC300148.1 | DANIELEWSKI et al., 2013 |
| AUS_RRP43315 | KC300147.1 | DANIELEWSKI et al., 2013 |
| AUS_RRP935815 | KC300146.1 | DANIELEWSKI et al., 2013 |
| AUS_RRP9453315 | KC300145.1 | DANIELEWSKI et al., 2013 |
| AUS_RRP54322 | KC300144.1 | DANIELEWSKI et al., 2013 |
| AUS_RRP42842 | KC300143.1 | DANIELEWSKI et al., 2013 |
| AUS_RRP21112 | KC300142.1 | DANIELEWSKI et al., 2013 |
| AUS_RRP18270 | KC300141.1 | DANIELEWSKI et al., 2013 |
| AUS_AC93 | KC333888.1 | DANIELEWSKI et al., 2013 |
| LZod45 | EU918768.1 | WU et al., 2009 |
| HPV11_ref | M14119.1 | DARTMANN et al., 1986 |
| SL_LP27 | FN870448.1 | MAVER et al., 2011 |
| SL_LP19 | FN870447.1 | MAVER et al., 2011 |
| SL_LP16 | FN870446.1 | MAVER et al., 2011 |
| SL_LP14 | FN870445.1 | MAVER et al., 2011 |
| SL_LP13 | FN870444.1 | MAVER et al., 2011 |
| SL_LP12 | FN870443.1 | MAVER et al., 2011 |
| SL_LP6 | FN870442.1 | MAVER et al., 2011 |
| SL_LP1 | FN870441.1 | MAVER et al., 2011 |
| SL_A418 | FN870497.1 | MAVER et al., 2011 |
| SL_A409 | FN870496.1 | MAVER et al., 2011 |
| SL_A383 | FN870495.1 | MAVER et al., 2011 |
| SL_A346 | FN870494.1 | MAVER et al., 2011 |
| SL_A345 | FN870493.1 | MAVER et al., 2011 |
| SL_A320 | FN870492.1 | MAVER et al., 2011 |
| SL_A297 | FN870491.1 | MAVER et al., 2011 |
| SL_A281 | FN870490.1 | MAVER et al., 2011 |
| SL_A260 | FN870489.1 | MAVER et al., 2011 |
| SL_A253 | FN870488.1 | MAVER et al., 2011 |
| SL_A250 | FN870487.1 | MAVER et al., 2011 |
| SL_A218 | FN870486.1 | MAVER et al., 2011 |
| SL_A205 | FN870485.1 | MAVER et al., 2011 |
| SL_A202 | FN870484.1 | MAVER et al., 2011 |
| SL_A201 | FN870483.1 | MAVER et al., 2011 |
| SL_A200 | FN870482.1 | MAVER et al., 2011 |
| SL_A197 | FN870481.1 | MAVER et al., 2011 |
| SL_A187 | FN870480.1 | MAVER et al., 2011 |
| SL_A107 | FN870479.1 | MAVER et al., 2011 |
| SL_CS123 | FN870478.1 | MAVER et al., 2011 |
| SL_CS93 | FN870477.1 | MAVER et al., 2011 |
| SL_CS58 | FN870476.1 | MAVER et al., 2011 |
| SL_CS20 | FN870475.1 | MAVER et al., 2011 |
| SL_A171 | FN870474.1 | MAVER et al., 2011 |
| SL_A161 | FN870473.1 | MAVER et al., 2011 |
| SL_A140 | FN870472.1 | MAVER et al., 2011 |
| SL_A136 | FN870471.1 | MAVER et al., 2011 |
| SL_A129 | FN870470.1 | MAVER et al., 2011 |
| SL_A128 | FN870469.1 | MAVER et al., 2011 |
| SL_A122 | FN870468.1 | MAVER et al., 2011 |
| SL_A101 | FN870467.1 | MAVER et al., 2011 |
| SL_A89 | FN870466.1 | MAVER et al., 2011 |
| SL_A86 | FN870465.1 | MAVER et al., 2011 |
| SL_A41 | FN870464.1 | MAVER et al., 2011 |
| SL_A63 | FN870463.1 | MAVER et al., 2011 |
| SL_A48 | FN870462.1 | MAVER et al., 2011 |
| SL_A35 | FN870461.1 | MAVER et al., 2011 |
| SL_A34 | FN870460.1 | MAVER et al., 2011 |
| SL_A18 | FN870459.1 | MAVER et al., 2011 |
| SL_A47 | FN870458.1 | MAVER et al., 2011 |
| SL_CAC11 | FN870449.1 | MAVER et al., 2011 |
| SL_CAC86 | FN870450.1 | MAVER et al., 2011 |
| SL_CAC246 | FN870451.1 | MAVER et al., 2011 |
| SL_CAC256 | FN870452.1 | MAVER et al., 2011 |
| SL_CAC266 | FN870453.1 | MAVER et al., 2011 |
| SL_CAC321 | FN870454.1 | MAVER et al., 2011 |
| SL_CAC336 | FN870455.1 | MAVER et al., 2011 |
| SL_CAC346 | FN870456.1 | MAVER et al., 2011 |
| SL_CAC372 | FN870457.1 | MAVER et al., 2011 |
| SL_A4 | FN870440.1 | MAVER et al., 2011 |
| SL_A51 | FN870439.1 | MAVER et al., 2011 |
| SL_A32 | FN870438.1 | MAVER et al., 2011 |
| SL_A57 | FN870437.1 | MAVER et al., 2011 |
| SL_A50 | FN870436.1 | MAVER et al., 2011 |
| SL_M3 | FN870435.1 | MAVER et al., 2011 |
| BR_LP25 | KC285862.1 | DE MATOS et al., 2013 |
| BR_LP19 | KC285861.1 | DE MATOS et al., 2013 |
| BR_LP20 | KC285860.1 | DE MATOS et al., 2013 |
| BR_LP24 | KC285859.1 | DE MATOS et al., 2013 |
| BR_LP23 | KC285858.1 | DE MATOS et al., 2013 |
| BR_LP22 | KC285857.1 | DE MATOS et al., 2013 |
| BR_LP21 | KC285856.1 | DE MATOS et al., 2013 |
| AUS_RRP76510 | KC329893.1 | DANIELEWSKI et al., 2013 |
| AUS_RRP75380 | KC329892.1 | DANIELEWSKI et al., 2013 |
| AUS_RRP63871 | KC329891.1 | DANIELEWSKI et al., 2013 |
| AUS_RRP45148 | KC329890.1 | DANIELEWSKI et al., 2013 |
| AUS_RRP57867 | KC329889.1 | DANIELEWSKI et al., 2013 |
| AUS_RRP49363 | KC329888.1 | DANIELEWSKI et al., 2013 |
| AUS_RRP33715 | KC329887.1 | DANIELEWSKI et al., 2013 |
| AUS_RRP23046 | KC329886.1 | DANIELEWSKI et al., 2013 |
| AUS_RRP65750 | KC329885.1 | DANIELEWSKI et al., 2013 |
| AUS_C410 | KC329874.1 | DANIELEWSKI et al., 2013 |
| AUS_AC100 | KC329873.1 | DANIELEWSKI et al., 2013 |
| AUS_AC68 | KC329872.1 | DANIELEWSKI et al., 2013 |
| AUS_GW0842L | KC329875.1 | DANIELEWSKI et al., 2013 |
| AUS_GW2481B | KC329877.1 | DANIELEWSKI et al., 2013 |
| AUS_GW2486A | KC329878.1 | DANIELEWSKI et al., 2013 |
| AUS_GW4028N1 | KC329879.1 | DANIELEWSKI et al., 2013 |
| AUS_GW5176F | KC329880.1 | DANIELEWSKI et al., 2013 |
| AUS_GW9384R | KC329882.1 | DANIELEWSKI et al., 2013 |
| AUS_GW9394B | KC329883.1 | DANIELEWSKI et al., 2013 |
| AUS_GW9399A | KC329884.1 | DANIELEWSKI et al., 2013 |
| AUS_GW0847K1 | KC329876.1 | DANIELEWSKI et al., 2013 |
| AUS_GW5350K | KC329881.1 | DANIELEWSKI et al., 2013 |
